# Necrological

**Published:** 1880-10

**Authors:** 


### NECROLOGICAL.

"Death is the crown of life:
Were death denied, poor man would live in vain."

Died on the 15th day of September, 1880, at Wadley, Georgia, Rev. William Hauser, M. D., aged about 70 years. Dr. Hauser was Professor of Physiology in Oglethorpe Medical College, for two years preceding the civil war, and the warm personal friendship that began with our association as colleagues in that institution, knew no abatement to the day of his death, but only increased and strengthened as the years rolled on. One of the best friends we have in Baltimore, became such through his disinterested kindness and his thoughtfulness for our welfare and happiness. We can safely say, we never knew a truer nor a better man. Whilst modest as a woman, he was firm and uncompromising in the maintenance of the right. Thoroughly cultivated and eloquent, he won the hearts of his students, and there are many physicians who listened to his grand and noble utterances now scattered over the vast South and South-West, whose eyes will moisten though all unused to tears, on learning of his demise.

"Green be the turf above thee,
Friend of my better days."

